# Outcome after cytoreductive surgery combined with hyperthermic intrathoracic chemotherapy in patients with secondary pleural metastases

**DOI:** 10.3389/fonc.2023.1259779

**Published:** 2023-11-28

**Authors:** Mohamed Hassan, Julia Zimmermann, Severin Schmid, Bernward Passlick, Julia Kovács, Rudolf Hatz, Hauke Winter, Laura V. Klotz, Martin E. Eichhorn, Till Markowiak, Karolina Müller, Gunnar Huppertz, Michael Koller, Hans-Stefan Hofmann, Michael Ried

**Affiliations:** ^1^Department of Thoracic Surgery, Medical Center—University of Freiburg, Freiburg im Breisgau, Germany; ^2^Faculty of Medicine, University of Freiburg, Freiburg im Breisgau, Germany; ^3^Department of Thoracic Surgery, Ludwig-Maximilians-University of Munich, Asklepios Lung Clinic, Gauting, Germany; ^4^Department of Thoracic Surgery, Thoraxklinik, University Hospital Heidelberg, Heidelberg, Germany; ^5^Translational Lung Research Center (TLRC) Heidelberg, Member of the German Center for Lung Research (DZL), Heidelberg, Germany; ^6^Department of Thoracic Surgery, University Hospital Regensburg, Regensburg, Germany; ^7^Center for Clinical Studies, University Hospital Regensburg, Regensburg, Germany; ^8^Department of Thoracic Surgery, Hospital Barmherzige Brüder Regensburg, Regensburg, Germany

**Keywords:** HITOC, cytoreductive surgery, pleural metastases, non-small cell lung cancer, hyperthermic intrathoracic chemotherapy

## Abstract

**Background:**

The role of cytoreductive surgery combined with hyperthermic intrathoracic chemotherapy (CRS+HITOC) for patients with secondary pleural metastases has scarcely been investigated.

**Patients and Methods:**

We conducted a retrospective, multicentre study investigating the outcome of CRS+HITOC for 31 patients with pleural metastases from different primary tumours in four high-volume departments of thoracic surgery in Germany. The primary endpoint was overall survival (OS). Secondary endpoints included postoperative complications and recurrence/progression-free survival (RFS/PFS).

**Results:**

The primary tumour was non-small cell lung cancer in 12 (39%), ovarian cancer in 5 (16%), sarcoma in 3 (10%), pseudomyxoma peritonei in 3 (10%), and others in 8 (26%) patients. A macroscopic complete resection (R/1) could be achieved in 28 (90%) patients. Major postoperative complications as classified by Clavien-Dindo (III-V) were observed in 11 (35%) patients. The postoperative mortality rate was 10% (n=3). A total of 13 patients received additive chemotherapy (42%). The median time of follow up was 30 months (95% CI = 17– 43). The median OS was 39 months (95% CI: 34-44 months) with 1-month, 3-month, 1-, 3-, and 5-year survival estimates of 97%, 89%, 77%, 66%, and 41%. There was a significantly prolonged OS in patients who received additive chemotherapy compared to patients with only CRS+HITOC (median OS 69 *vs* 38 months; p= 0.048). The median RFS was 14 months (95% CI: 7-21 months).

**Conclusions:**

We observed that CRS+HITOC is a feasible approach with reasonable complications and prolonged survival as a part of multimodal concept for highly selected patients with secondary pleural metastases.

## 1 Introduction

For many tumours, development of synchronous or metachronous metastases correlates with poor survival (1). This is particularly true in the case of secondary pleural metastases, where local therapy options are significantly limited and only palliative treatment concepts in the sense of systemic chemotherapy or best supportive care can be offered. Either pleurodesis or an indwelling permanent pleural catheter can be performed to control the usually present malignant pleural effusion and to reduce effusion-related symptoms (2). A multimodal treatment approach combining systemic therapies with local therapies might lead to the best outcome for selected patients (3). However, in view of the advanced stage and the consequent palliative situation, it is important to identify suitable patients for multimodal therapy and to determine the best possible local pleural treatment. Additional intracavitary therapy like using hyperthermic intrathoracic chemotherapy (HITOC) after cytoreductive surgery (CRS) has been shown to be a feasible and safe treatment approach for patients with pleural malignancies, especially for malignant pleural mesothelioma or thymic tumours with pleural spread (4, 5). The combination of CRS and HITOC within the multimodal treatment of patients with secondary pleural metastases of other primary tumours has been evaluated only in few studies so far and included in particular pleural spread of lung cancer, ovarian cancer and pseudomyxoma (6–9). The aim of CRS before HITOC is to achieve macroscopic complete tumour resection (MCR) (10). The residual tumour cells are then treated with hyperthermic chemotherapeutical agents to improve local tumour control and to delay tumour recurrence with at best prolonged survival rates (11). Despite high concentrations of chemotherapeutic agents in the thoracic cavity, systemic side effects are fewer than during systemic treatment due to reduced intrathoracic absorption of the chemotherapy (12). Objective of this study was to investigate the feasibility and the outcome of radical local surgery including intracavitary chemotherapy in patients with secondary pleural metastases. Therefore, a selected and heterogenous subgroup of patients from the German HITOC-Study who underwent CRS+HITOC were analysed.

## 2 Patients and methods

### 2.1 Study design

In this retrospective multicentre study, clinical outcomes of patients with secondary pleural metastases after radical local surgery including intracavitary chemotherapy were analysed. Four high-volume departments of thoracic surgery in Germany collected data in the “German HITOC-Study” funded by the Deutsche Forschungsgemeinschaft – DFG, German Research Association (DFG, RI2905/3-1). The study group has already published first results about HITOC after CRS for pleural malignancies in general and for thymic tumours with pleural spread (5, 13, 14). Now, the focus will be on secondary pleural metastases and its surgical treatment combined with HITOC. The trial is registered in the German Registry of Clinical Studies (DRKS-ID: DRKS00015012). The approval of the ethics committee of the University of Regensburg (reference number: 18-1119-104) and of the ethics committees of the respective participating centres were obtained. All patients underwent elective CRS+HITOC within one surgery and in one of the four participating departments for thoracic surgery between January 2008 and December 2019 (13). The existing database was supplemented by an additional collection of clinical and follow-up data, which were updated until November 2021.

### 2.2 Definitions of variables and data collection

This subanalysis included all patients of the complete study sample (n= 350) with secondary pleural metastases (n= 31). Additive chemotherapy was defined as either induction chemotherapy and/or adjuvant chemotherapy. Postoperative complications were documented according to the Clavien-Dindo classification (15). The beginning of primary therapy was defined as date of CRS+HITOC if no induction chemotherapy was applied, or if induction chemotherapy was applied, as the date of first application of induction chemotherapy.

### 2.3 Endpoints

Primary endpoint of the study was Overall survival (OS) after CRS+HITOC. Secondary endpoints included postoperative morbidity/mortality and Recurrence and progression-free survival (RFS/PFS). OS was defined as time from beginning of the primary therapy until death from any cause. Recurrence and progression were defined as documented intrathoracic, ipsilateral and/or contralateral tumour detection by cytology/histology and/or imaging. RFS/PFS was defined as time from beginning of the primary therapy until first objective tumour recurrence/progression or death from any cause, which ever occurred first.

### 2.4 Statistical analysis

Descriptive analyses were done for demographic and baseline characteristics using frequency (n), percentage (%), mean (m), standard deviation (SD), median (med) and interquartile range (IQR). Median follow-up time was calculated using the reversed Kaplan-Meier method. OS and RFS/PFS were analysed by the Kaplan-Meier estimator. In univariable analyses (Log-Rank tests), OS and PFS/RFS were compared between cisplatin dosage (low dose ≤ 125 mg/m² BSA; high dose > 125 mg/m² BSA), chemotherapeutical agent (cisplatin alone *vs* cisplatin+doxorubicin), resection status, and additive chemotherapy. The estimates for the probability of surviving were presented graphically in Kaplan-Meier survival curves as well as for the specific time points: 1-month, 3-month, 1-year, 3-year, and 5-year survival rate. Moreover, the median survival time with 95% CI were presented when possible. All statistical analyses were conducted using the software package SPSS (version 26 or higher).

## 3 Results

### 3.1 Patient characteristics

Of the 31 included patients the mean age at the time of surgery was 50.3 ± 14.8 years. The primary pathology was non-small cell lung cancer (NSCLC) in 12 (39%), ovarian cancer in five (16%), sarcoma in three (10%), pseudomyxoma peritonei in three (10%), and others in eight (26%) patients. Pleural metastases were diagnosed with thoracoscopy and pleural biopsy in 18 (58%) patients (**Table 1**).

**Table 1:** Patient characteristics.

	Study population n= 31
**Sex (n, %) ** female male	20 (64.5%) 11 (35.5%)
**Age (mean; SD) [years]**	50.3 (14.8)
**BMI (mean; SD) [kg/m²]**	25.1 (5.1)
**Karnofsky-index (n, %) ** 70% 80% 90% 100% missing	2 (6.5%) 7 (22.6%) 16 (51.6%) 5 (16.1%) 1 (3.2%)
**ECOG-status (n, %) ** 0 1 missing	21 (67.7%) 9 (29.0%) 1 (3.2%)
**Confirmation of pleural tumour diagnosis** (multiple answers possible) **(n, %) ** VATS CT-biopsy bronchoscopy pleural punctate not specified	18 (58.1%) 3 (9.7%) 2 (6.5%) 5 (16.1%) 5 (16.1%)
**Pleural tumour manifestation (n, %) ** first diagnosis recurrence	26 (83.9%) 5 (16.1%)
**Primary pathology ** Non-small cell lung cancer Ovarian cancer Sarcoma Pseudomyxoma peritonei Other pathology - Breast cancer - Prostate cancer - Cecum cancer - Adenoid cystic carcinoma of the parotid gland - Malignant Peripheral Nerve Sheath Tumor - Sinonasal Squamous Cell Carcinoma - neuroendocrine carcinoma	12 (38.7%) 5 (16.1%) 3 (9.7%) 3 (9.7%) 8 (25,8%)

BMI, body mass index; VATS, video-assisted thoracic surgery; CT, computed tomography.

### 3.2 Operative and systemic treatments

All operations were done with a thoracotomy. Pleurectomy and decortication (P/D) was performed in five (16%), extended pleurectomy and decortication (eP/D) in 25 (81%), and extrapleural pneumonectomy (EPP) in one (3%) patient. Extended resections included additional chest wall resection in seven (23%) and anatomical lung resection in eight (26%) patients when appropriate due to tumour infiltration. A MCR (R0/1) could be achieved in 28 (90%) patients. HITOC with cisplatin only was performed in 22 (71%) and in combination with doxorubicin in nine (29%) patients. The median duration of surgery was 350.6 ± 71.6 minutes. There was no HITOC associated intraoperative complication. Additive chemotherapy was conducted in 13 patients (42%), which included neoadjuvant and/or adjuvant chemotherapy. **Table 2** summarizes operative data and treatment details.

**Table 2:** Operative data and treatment details.

	Study population n= 31
**Side of operation (n, %) ** left right	17 (54.8%) 14 (45.2%)
**Resection method (n, %) ** P/D eP/D EPP	5 (16.1%) 25 (80.6%) 1 (3.2%)
**Resection of** (multiple answers possible) **(n, %) ** diaphragm pericardium chest wall lung (anatomical)	19 (61.3%) 10 (32.3%) 7 (22.6%) 8 (25.8%)
**Alloplastic reconstruction/replacement of (n, %) ** diaphragm pericardium	7 (22.6%) 5 (16.1%)
**Intraoperative resection status (n, %) ** R0/1 (MCR) R2	28 (90.3%) 3 (9.7%)
**Duration of (median, IQR) [min] ** CRS + HITOC HITOC	345 (296 - 408) 60 (60 - 70)
**Cisplatin dose (n, %) ** low dose (≤ 125 mg/m² BSA) high dose (>≤ 125 mg/m² BSA)	13 (41.9%) 18 (58.1%)
**Cisplatin agent (n, %) ** Cisplatin Cisplatin + Doxorubicin	22 (71.0%) 9 (29.0%)
**Intraoperative complication (n, %)**	0
**Complications HITOC (n, %)**	0
**Additive chemotherapy (n, %)** neoadjuvant chemotherapy adjuvant chemotherapy	13 (41.9%) 7 (22.6%) 7 (22.6%)
**Adjuvant radiotherapy (n, %) ** yes no missing data	2 (6.5%) 25 (80.6%) 4 (12.9%)

CRS, cytoreductive surgery; eP/D, extended pleurectomy/decortication; EPP, extrapleural pneumonectomy; HITOC, hyperthermic intrathoracic chemotherapy; MCR, macroscopic complete resection; P/D, pleurectomy/decortication.

### 3.3 Postoperative course and complications

Major postoperative complications as classified by Clavien-Dindo (III-V) were observed in eleven patients (35%; **Table 3**). In total, six patients (19%) required surgical revision in consequence of hemothorax, chylothorax, pleural empyema, or prolonged air leak. Postoperative acute renal failure was observed in four patients (13%). Only one patient required postoperative dialysis. The postoperative mortality rate was 10% (n=3). The median ICU length of stay was two days (IQR: 1-4) and the median hospital length of stay was 15 days (IQR: 11 to 28).

**Table 3:** Postoperative complications.

	Study population n=31
**Postoperative complications (n, %) ** no yes/Clavien-Dindo classification I II IIIa IIIb V	15 (48.4%) 16 (51.6%) 2 (6.5%) 3 (9.7%) 3 (9.7%) 5 (16.1%) 3 (9.7%)
**Surgical revision (n, %) ** no yes hematothorax pleural empyema chylothorax parenchymal/bronchial fistula	25 (80.6%) 6 (19.4%) 2 (6.5%) 1 (3.2%) 1 (3.2%) 2 (6.5%)
**Prolonged ventilation >24h (n, %)**	3 (9.7%)
**Prolonged parenchyma fistula (air leak > 7 days) (n, %)**	1 (3.2%)
**Post-operative atrial fibrillation (n, %)**	2 (6.5%)
**Post-operative sepsis (n, %)**	2 (6.5%)
**Post-operative pneumonia (n, %)**	2 (6.5%)
**Post-operative pulmonary embolism (n, %)**	0
**Post-operative renal insufficiency (n, %)**	4 (12.9%)
**Post-operative dialysis (n, %)**	1 (3.2%)
**Duration of ICU stay [days] (median, IQR)**	2 (1 - 4)
**Duration of hospitalization [days] (median, IQR)**	15 (11 - 28)
**In-hospital mortality (n, %)**	3 (9.7%)

### 3.4 Survival analysis

**Figure 1:**
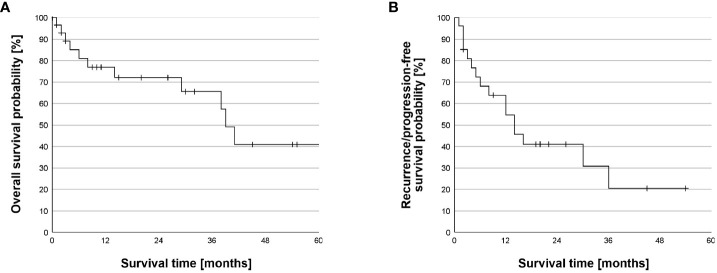
Overall Survival and recurrence/progression-free survival. **(A)** Kaplan-Meier survival analysis of overall survival (OS). The median OS was 39 months (95% CI: 34-44 months). The 1-month, 3-month, 1-, 3-, and 5-year survival estimates were 97%, 89%, 77%, 66%, and 41%. Survival time was truncated at 60 months after beginning of the primary therapy. One patient died 69 months after beginning of the primary therapy. **(B)** Kaplan-Meier survival analysis of recurrence/progression-free survival (RFS/PFS). The median RFS/PFS was 14 months (CI 95%: 6.9-21.1 months). The 1-month, 3-month, 1-, and 3-year survival estimates of 96%, 81%, 55%, and 21%.

**Figure 2:**
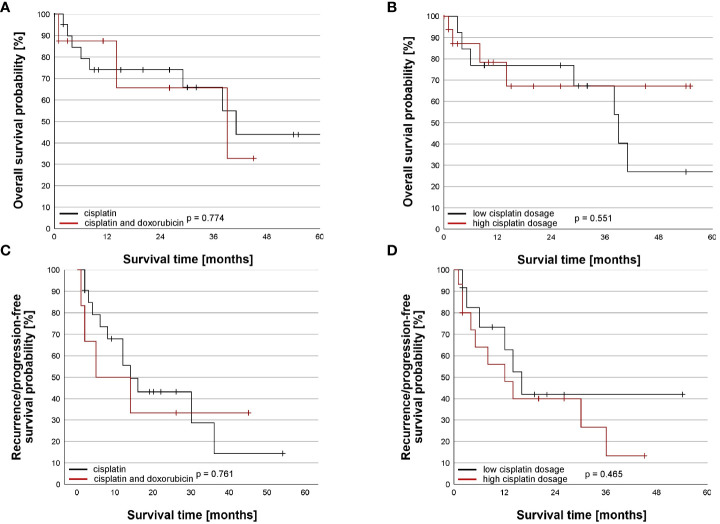
Impact of chemotherapeutic regime on the overall and recurrence/progression-free survival. **(A)** Comparison of overall survival (OS) between chemotherapeutical agent. OS did not differ between patients receiving cisplatin alone (n = 22) and cisplatin and doxorubicin (n= 8) (p=0.774). Survival time was truncated at 60 months after beginning of the primary therapy. One patient with cisplatin alone died 69 months after beginning of the primary therapy. **(B)** Comparison of overall survival (OS) between cisplatin dosage. OS did not differ between patients receiving low dose cisplatin (n = 13) and high dose cisplatin (n=17) (p=0.551). Survival time was truncated at 60 months after beginning of the primary therapy. One patient with low cisplatin dosage died 69 months after beginning of the primary therapy. **(C)** Comparison of recurrence/progression-free survival (RFS/PFS) between chemotherapeutical agent. RFS/PFS did not differ between patients receiving cisplatin alone (n = 21) and cisplatin and doxorubicin (n= 6) (p=0.761). **(D)** Comparison of recurrence/progression-free survival (RFS/PFS) between cisplatin dosage. RFS/PFS did not differ between patients receiving low dose cisplatin (n = 12) and high dose cisplatin (n= 15) (p=0.465).

**Figure 3:**
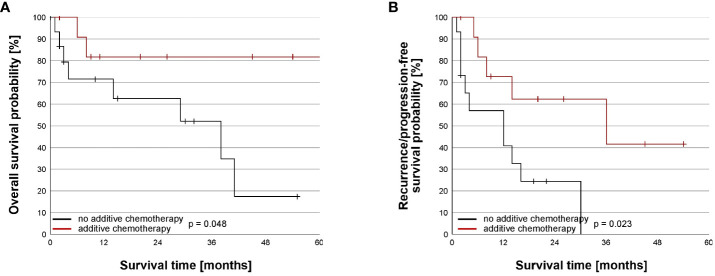
Impact of additive chemotherapy on the overall and recurrence/progression-free survival. **(A)** Overall survival (OS) was improved in patients receiving additive chemotherapy (n = 12, med = 69 months, 95% CI = NA) compared to patients without additive chemotherapy (n= 16, med = 38 months, 95% CI = 9, 67) (p=0.048). Survival time was truncated at 60 months after beginning of the primary therapy. One patient with additive chemotherapy died 69 months after beginning of the primary therapy. **(B)** Recurrence/progression-free survival (RFS/PFS) was improved in patients receiving additive chemotherapy (n = 12, med = 36 months, 95% CI = 0, 77) compared to patients without additive chemotherapy (n= 15, med = 12 months, 95% CI = 0, 25) (p=0.023).

Due to missing data, one patient had to be excluded from the survival analysis (**Table 4**). The median time of follow up was 30 months (95% CI = 17– 43, n = 30). The median OS was 39 months (95% CI: 34-44) with 1-month, 3-month, 1-, 3-, and 5-year survival estimates of 97%, 89%, 77%, 66%, and 41% (**Figure 1A**). Adding doxorubicin to cisplatin for HITOC was not associated with improved OS (p= 0.774, **Figure 2A**). The use of high dose cisplatin did not lead to prolonged OS compared to low dose cisplatin (p= 0.551; **Figure 2B**). The median OS improved significantly in patients who received additive chemotherapy compared to patients with only CRS+HITOC (median OS 69 *vs* 38 months; p= 0.048, n = 28; **Figure 3A**). Macroscopic incomplete resection (R2) was performed only in two (6%) patients and was associated with significantly decreased OS compared to 28 patients who had macroscopic complete resection (R0/1) (median OS 3 *vs* 41 months, p= 0.023). No evidence of disease recurrence or progress was documented in 18 patients (58%). Locoregional recurrence was observed in five (16%) and distant metastases in five (16%) patients. Due to missing data about tumour recurrence/progression status and date of start of primary therapy, four patients were excluded from analyses. The median RFS/PFS for the included patients (n= 27) was 14 months (95% CI: 7-21 months; **Figure 1B**). The use of high dose cisplatin or adding doxorubicin to cisplatin was not associated with improved RFS/PFS (p values > 0.050; **Figures 2C, D**). Patients who received additive chemotherapy showed an advantage in RFS/PFS compared to only CRS+HITOC (36 *vs* 12 months; p= 0.023; **Figure 3B**). Macroscopic incomplete resection (R2) was performed only in one patient and was associated with significantly decreased RFS/PFS compared to 26 patients who had macroscopic complete resection (R0/1) (median OS 3 *vs* 41 months, p= 0.064). A subgroup analysis of twelve patients with pleural metastasis of lung carcinoma (stage IV) showed a 3- month, 1-year and 3-year OS of 100%, 80% and 80%, Median PFS/RFS was 30 months (95% CI= 0-71) with estimated survival times at 3-month, 1-year and 3-year of 100%, 60% and 30%.

**Table 4:** Kaplan-Meier analysis of overall survival (OS) and recurrence/progression-free survival (RFS/PFS).

		n	n events	1-month survival	3-month survival	1-year survival	3-year survival	5-year survival	p value
OS*		30	12	0.97	0.89	0.77	0.66	0.41	
cisplatin dosage	low	13	8	0.92	0.92	0.77	0.67	0.27	0.551
	high	17	4	0.94	0.87	0.78	0.67	–	
chemotherapeutical agent	cisplatin alone	22	9	0.95	0.90	0.74	0.66	0.44	0.774
	cisplatin + doxorubicin	8	3	0.88	0.88	0.88	0.66	–	
resection status	R0/1	28	11	0.96	0.93	0.80	0.68	0.43	0.023
	R2	2	1	1.00	0.00				
additive chemotherapy**	no	16	8	0.93	0.79	0.72	0.52	–	0.048
	yes	12	3	1.00	1.00	0.82	0.82	0.82	
Overall RFS/PFS`***		27	16	0.96	0.81	0.55	0.21	–	
cisplatin dosage	low	12	6	0.91	0.83	0.63	0.42	–	0.465
	high	15	10	0.93	0.80	0.48	0.13	–	
chemotherapeutical agent	cisplatin alone	21	12	1.00	0.85	0.56	0.14	–	0.761
	cisplatin + doxorubicin	6	4	0.83	0.67	0.50	0.33	–	
resection status	R0/1	26	15	0.96	0.85	0.57	0.22	–	0.064
	R2	1	1	1.00	0.00				
additive chemotherapy	no	15	11	0.93	0.65	0.41	0.00		0.023
	yes	12	5	1.00	1.00	0.62	0.42	–	

Event was defined as time from beginning of the primary therapy until death from any cause in case of OS or time from beginning of the primary therapy until first objective tumour recurrence/progression or death from any cause, which ever occurred first in case of RFS/PFS.

*1 patient was excluded due to missing data.

**2 patients were excluded due to missing data.

***4 patients were excluded due to missing data.

## 4 Discussion

Despite advances in systemic therapy for metastatic cancers, the prognosis of secondary pleural metastases remains poor with median survival times from four to nine months (16–19). The main finding of our study was a prolonged survival in selected patients with secondary pleural metastases after CRS+HITOC as a part of multimodal treatment approach with median overall survival of 39 months and median RFS/PFS of 14 months. Retrospective studies showed an encouraging survival of patients with pleural mesothelioma or thymus tumours with pleural involvement, who underwent a multimodal treatment approach including local interventions with CRS+HITOC (5, 11, 20). However, there is paucity of studies investigating the outcome of CRS+HITOC in patients with secondary pleural metastases as a part of multimodal concept. Therefore, the role of local interventions is still limited to reduce the symptoms of pleura effusions with an indwelling pleural catheter or pleurodesis (2, 17). CRS+HITOC can be performed after interdisciplinary discussion in the context of a multimodal approach in highly selected patients with an adequate general condition. Previous analyses confirmed the feasibility and safety of this combined surgical approach (14). Our sub-analysis also showed a reasonable major postoperative complication rate of 35% and a postoperative in-hospital mortality of 10%. We could not retrospectively provide the cause of postoperative mortality in the three patients. In addition to clinical (tumour stage, thoracic tumour spread) and functional parameters (above all ECOG), the decision on this radical procedure should also depend in particular on the histology of the primary tumour. We recommend a complete staging including mediastinal staging and PET-CT, however we could not retrospectively provide this data. Our study population included pleural metastases of various histological entities as mentioned above, but 12 patients (39%) in our study underwent CRS+HITOC for pleural metastases secondary to non-small cell lung cancer with encouraging survival rates of 80% OS after 3 years. Shigemura et al. showed a mean survival of 19 months in five patients with lung cancer and pleural metastases after a 2-step approach consisting of thoracoscopic intrathoracic chemotherapy followed by radical cytoreductive surgery in the form of an EPP (8). In our cohort, lung-sparing eP/D was the most performed technique of CRS (80.6%) and only one patient underwent EPP. EPP should be avoided when possible due to the high risk of early and late postoperative complications (21). However we could not investigate this factor as only one patient underwent EPP in our study.

In a systematic review investigating 21 patients who received CRS+HITOC for lung cancer with pleural metastases from four retrospective studies, the median survival was 18 months, which represents an encouraging result compared to the poor prognosis known in the literature with median survival of only 4 months (7, 22–24). However, these results should be cautiously interpreted due to the limited number of included patients. A previous study showed that about 60% of patients with pleural metastases are secondary to extra thoracic malignancies, with breast cancer as the most common primary with incidence about 25% followed by lymphoma (10%), ovarian cancer (5%), and intraabdominal malignancies (5%) (18). The complete surgical cytoreduction of metastatic ovarian cancer in the abdominal cavity was shown to be an independent prognostic factor, however the role of CRS in thoracic cavity has scarcely been reported (25, 26). Boerner et al. showed a promising survival in patients with ovarian cancer with pleural metastases after combined abdominal and thoracic CRS (27). The use of HITOC without CRS showed to be helpful for relieving the symptoms of pleural effusion in patients with metastatic ovarian cancer (28). Nikiforchin et al. showed promising results of CRS+HITOC for pleural metastases of peritoneal surface malignancies with 1-, 3-, and 5-year OS of 93%, 68%, and 68% with a median follow up of 22 months. The CRS+HITOC was performed either as a separate procedure via thoracotomy or as a part of the CRS+HIPEC through the diaphragm. The major postoperative complication rate was 20% for the combined CRS+HIPEC/HITOC and 13% for CRS+HITOC. In our study we reported a major postoperative complication rate of 35%. The higher incidence of major postoperative complication in our cohort may be associated to the extended resections including anatomical lung resections (26%) and chest wall resections (23%) which were not performed in the study of Nikiforchin et al. (3). For the various histological entities apart from non-small cell lung cancer in this study population, we were not able to perform any meaningful survival analyses due to the different entities with correspondingly low case numbers, so that studies with larger cohorts must be awaited here. Unfortunately, this is difficult to implement due to the specific indications (3). Likewise, clear recommendations for the selection of the intrathoracic chemotherapeutic agent and its dosage are still lacking. So far, only the data on HITOC in MPM and pleural metastatic thymic tumours can be used as a guide (9). To date, the most commonly used substances are cisplatin, doxorubicin and mitomycin C (9). Sugarbaker et al. showed a persistent high intrapleural concentration of doxorubicin and mitomycin C for patients undergoing HITOC and provided a pharmacological rationale for HITOC (12). We observed no advantage in the OS or RFS/PFS using high dose of cisplatin or adding doxorubicin. Based on our results, a decisive criterion for the preoperative indication seems to be the possibility of macroscopic complete resection. The impact of the resection status after CRS+HITOC for secondary pleural metastases were mostly not even investigated. Our results indicate that macroscopic complete resection could lead to a prolonged OS and RFS/PFS. These results reveal the importance of a careful selection of patients who might profit from CRS+HITOC. However, these findings should be interpreted with caution as this analysis included a very limited number of patients with macroscopic incomplete resection (two patients for OS and only one patient for RFS/PFS). Pleural metastases are usually a sign of systemic disease with poor prognosis. However, there is a trend of improvement of the survival in patients with pleural metastases comparing earlier with most recent studies, which could reflect the evolution of systemic treatment options (29, 30). Our results also support this assumption, as patients who received additive chemotherapy showed a significantly prolonged survival compared to patient who underwent only local therapy with CRS+HITOC. Furthermore, the CRS+HITOC in our study was an individual concept as a part of multimodal treatment in selected patients without evidence of others metastases. We recommend that all patients should be discussed preoperatively in an interdisciplinary tumour board, which should recommend this treatment on a case-by-case basis.

## 5 Limitations

The major limitations of our study were the retrospective nature, the absence of a comparative group and the heterogeneity of included patients. Retrospectively, we could not comprehend why some patients in our study did not receive any additional systemic chemotherapy for secondary pleural metastases. Another aspect which should be considered is the limited number of patients who underwent CRS+HITOC for secondary pleural metastases in four high volume departments for thoracic surgery in Germany over eleven years. This might reflect the high selection of patients and cannot lead to a definitive conclusion regarding the role of CRS+HITOC as a part of treatment of this condition. However, we present one of the largest series of patients who underwent this local, intracavitary treatment approach for pleural metastases which may help for better selection of patients until further studies are available.

## 6 Conclusions

CRS+HITOC for secondary pleural metastases is feasible and associated with reasonable postoperative morbidity and mortality. We were able to observe a prolonged survival in highly selected patients with secondary pleural metastases after CRS+HITOC as a part of a multimodality treatment concept, especially in patients with stage IV lung carcinoma. In our view, a strict indication for this surgical procedure is required, which should especially take into account the chance for MCR and the possibility of additive systemic therapy.

## Data availability statement

The raw data supporting the conclusions of this article will be made available by the authors, without undue reservation.

## Ethics statement

The studies involving humans were approved by the ethics committee of the University of Regensburg (reference number: 18-1119-104) and of the ethics committees of the respective participating centers were obtained. The studies were conducted in accordance with the local legislation and institutional requirements. The ethics committee/institutional review board waived the requirement of written informed consent for participation from the participants or the participants’ legal guardians/next of kin because Patient consent was waived due to the retrospective design of the study and collection of routine clinical data. Data was processed in a pseudonymised manner in accordance with European Union General Data Protection Regulation (EUGDPR) and Bavarian Hospital Law (BayKrG).

## Author contributions

MH: Investigation, Validation, Writing – original draft, Writing – review & editing. JZ: Investigation, Validation, Writing – original draft, Writing – review & editing. SS: Validation, Writing – review & editing, Conceptualization, Writing – original draft. BP: Conceptualization, Supervision, Writing – review & editing. JK: Investigation, Writing – original draft, Writing – review & editing. RH: Conceptualization, Supervision, Writing – review & editing. HW: Supervision, Writing – review & editing. LK: Investigation, Writing – review & editing. ME: Conceptualization, Writing – review & editing. TM: Investigation, Methodology, Resources, Writing – review & editing. KM: Data curation, Formal Analysis, Methodology, Project administration, Software, Writing – review & editing, Visualization. GH: Formal Analysis, Methodology, Software, Writing – review & editing. MK: Data curation, Formal Analysis, Methodology, Software, Writing – review & editing. HH: Conceptualization, Supervision, Writing – review & editing, Resources. MR: Conceptualization, Project administration, Writing – original draft, Writing – review & editing, Funding acquisition, Investigation, Methodology.
